# Genetic Diversity and Structure of Dalmatian Pyrethrum (*Tanacetum cinerariifolium* Trevir. /Sch./ Bip., Asteraceae) within the Balkan Refugium

**DOI:** 10.1371/journal.pone.0105265

**Published:** 2014-08-14

**Authors:** Martina Grdiša, Zlatko Liber, Ivan Radosavljević, Klaudija Carović-Stanko, Ivan Kolak, Zlatko Satovic

**Affiliations:** 1 Department of Seed Science and Technology, Faculty of Agriculture, University of Zagreb, Zagreb, Croatia; 2 Department of Botany, Division of Biology, Faculty of Science, University of Zagreb, Zagreb, Croatia; St. Petersburg Pasteur Institute, Russian Federation

## Abstract

Dalmatian pyrethrum (*Tanacetum cinerariifolium* Trevir. /Sch./ Bip.) is an outcrossing, perennial insecticidal plant, restricted to the eastern Adriatic coast (Mediterranean). Amplified fragment-length polymorphisms (AFLP) were used to investigate the genetic diversity and structure within and among 20 natural plant populations. The highest level of gene diversity, the number of private alleles and the frequency down-weighted marker values (*DW*) were found in northern Adriatic populations and gradually decreased towards the southern boundary of the species range. Genetic impoverishment of these southern populations is most likely the result of human-related activities. An analysis of molecular variance (AMOVA) indicated that most of the genetic diversity was attributed to differences among individuals within populations (85.78%), which are expected due to the outcrossing nature of the species. A Bayesian analysis of the population structure identified two dominant genetic clusters. A spatial analysis of the genetic diversity indicated that 5.6% of the genetic differentiation resulted from isolation by distance (IBD), while 12.3% of the genetic differentiation among populations followed the pattern of isolation by environmental distance (IBED). Knowledge of the genetic diversity patterns of the natural populations and the mechanism behind these patterns is required for the exploitation and possible conservation management of this endemic and economically important species.

## Introduction

Conservation of genetic diversity is essential for any species long-term survival [1], providing the ability to respond to selective pressures caused by both anthropogenic and natural environmental changes [2]. Patterns of genetic diversity and population structure are specifically shaped in response to many factors and are result of both past history and present processes [3]. Primarily, climatic oscillations during the Quaternary causing repeated restriction-recolonization events have left distinguishing signatures in modern species genetics [4]. These oscillations have had a considerable impact on the current distribution and genetic diversity in numerous animal and plant species [5]–[8] in which altitudinal and latitudinal range shifts were a likely response to intense climatic changes [9]–[10]. Detailed phylogeographical studies have revealed that three European peninsulas of the Mediterranean area (Apennines, Balkans and Iberia) have served as refugia for many temperate species and a postglacial recolonization source for central and northern Europe [3], [7], [9]–[11]. However, this traditional interpretation of southern European peninsulas as being single glacial refugia has been modified, and the model “refugia within refugia” was developed [10], [12]–[15]. Genetic divergence expressed as a rarity index (*DW*; frequency down-weighted marker values) as an indicator of the historical processes of a species [16] enables the distinction between old vicariance and recent dispersal. As a consequence of long-term isolation, populations in glacial refugia are characterized by high levels of *DW* values and genetic diversity [17], whereas populations that were established after the most recent glacial cycle show little genetic variation [18] as an outcome of successive founder events during postglacial colonization [6], [9]. However, zones of secondary contact of divergent lineages can also exhibit high levels of genetic diversity [11], [19].

The Balkan Peninsula itself represents a unique ecological and biogeographical phenomenon in Europe possessing the tremendous number of plant and animal species and pronounced richness in endemic taxa as well. This remarkable diversity is the result of the variety of its regions, complex geological history, and interactions between populations, species and ecosystems [20]. In the last decade various phylogeographic and molecular studies have been conducted in this area with the aim of elucidating the genetic relationships of its flora and fauna [10], [21]–[25].

It is widely acknowledged that geographical and environmental factors can also play significant roles in the modeling of species-specific inter- and intra-population genetic structure. Spatially separated populations may experience isolation by distance (IBD) phenomena in which geographical barriers and physical distances cause restricted pollen and seed dispersal, i.e., gene flow [26]–[30]. Greater genetic divergence between populations inhabiting different environments suggests that various present and/or past ecological conditions may significantly impact the genetic differentiation of local populations [31]–[32], forming an isolation by environmental distance (IBED) pattern [33]. A significant positive partial correlation, after eliminating the effect of geographic distance, indicates the contribution of climatic gradients to patterns of genetic divergence [33]–[34].

Nevertheless, species are greatly affected by direct or indirect human activities including population fragmentation and overharvesting pressures, all resulting in loss of genetic diversity and the alteration of their population genetic structure [35]. Moreover, founder effects and conscious or unconscious human selections for high yield and quality through cultivation of wild plants likewise impose profound impacts on the genetic diversity patterns by narrowing genetic base of the plant material [36]–[38].

For our investigation we have chosen Dalmatian pyrethrum (*Tanacetum cinerariifolium* Trevir. /Sch./ Bip.), an insecticidal perennial plant. Dalmatian pyrethrum is an outcrossing diploid (2n = 18) [39], self-incompatible and thermophytic plant species of the family Asteraceae [40]. This species is restricted to the eastern coast of the Adriatic Sea, and its distribution range expands from the coastal region to 200 m.a.s.l., but in some cases, it is also present in higher mountainous Mediterranean zones at elevation above 500 m.a.s.l. Continuously distributed populations of Dalmatian pyrethrum can be found in extremely degraded habitats with shallow rocky soils. In Croatia, abundant populations can be found in the southern parts of the Istrian Peninsula (Premantura), the Kvarner islands (Krk, Cres and Lošinj), the Velebit and Biokovo mountains and along the Dalmatian coastal region and its islands (Brač, Hvar, Biševo, Vis, Korčula, Lastovo and Mljet) [41]. Apart from Croatia distribution range expands to the southern parts of Bosnia and Herzegovina, and coastal regions of Montenegro and Albania [42]. Plants produce the natural insecticide pyrethrin, which attacks the nervous systems of the insects causing a knock-down effect and death [43].

The cultivation and use of Dalmatian pyrethrum and its products have a documented history in Croatia. The dust from grinding dried flowers has traditionally been used in Croatian households and agriculture [44]. Originally, flowers were excessively gathered from the wild resources; however, due to the high demand for plant material, cultivation began in 1850s near Dubrovnik and rapidly expanded along the Dalmatian coastal region and the islands [45]. The first fields were established with seeds gathered from the wild. Dalmatian pyrethrum was primarily reproduced through vegetative splits and later by directly sowing the seeds [46]. It was grown in the middle and southern parts of Dalmatia, including Zadar, Biograd, Šibenik, Split, Hvar, Brač, Makarska, Korčula and Dubrovnik, as well as the northern Adriatic islands Krk and Cres [44]. In 1914, Croatia became the leading producer with high quality plant material. The highest yield was achieved in 1926 with approximately 1,359 tons of dried pyrethrum flowers [46].

The goal of this study was to analyze the genetic diversity and structure within and among 20 populations of Dalmatian pyrethrum across their geographical range in Croatia and to determine impact of (*i*) Pleistocene climatic oscillations, (*ii*) geographic (IBD) and (*iii*) environmental isolation (IBED), and (*iiii*) human activities through overexploitation and cultivation on the obtained pattern of genetic diversity. AFLP markers have found the widest application in assessing the variability and genetic structure of populations in a variety of plants [47]–[53] and once again have proven to be invaluable tools in describing the former. Despite its historical significance and value, the genetic diversity and structure of Dalmatian pyrethrum has never been investigated; therefore, this study using AFLP markers represents the first such attempt. Dalmatian pyrethrum is nationally strictly protected by The Croatian Ordinance on the proclamation of protected and strictly protected wild taxa (Official Gazette 99/09; http://narodne-novine.nn.hr/clanci/sluzbeni/dodatni/403046.pdf). Today, as an endemic species, Dalmatian pyrethrum is threatened by anthropogenic habitat loss and degradation related to factors such as urbanization and habitat conversion; therefore, the need and importance of these species’ genetic resources protection must be recognized even more. The gathered information could be of utmost importance for future activities regarding the conservation and breeding programs of this endemic and economically important species.

## Materials and Methods

### Ethics statement

Collecting of all samples from natural habitats in Croatia including Nature Park Biokovo was permitted by the authority of Directorate for Nature Protection of the Ministry of Culture of the Republic of Croatia (532-08-01/6-06-02; 07.07.2006).

### Sampling of plant populations

**Figure 1:**
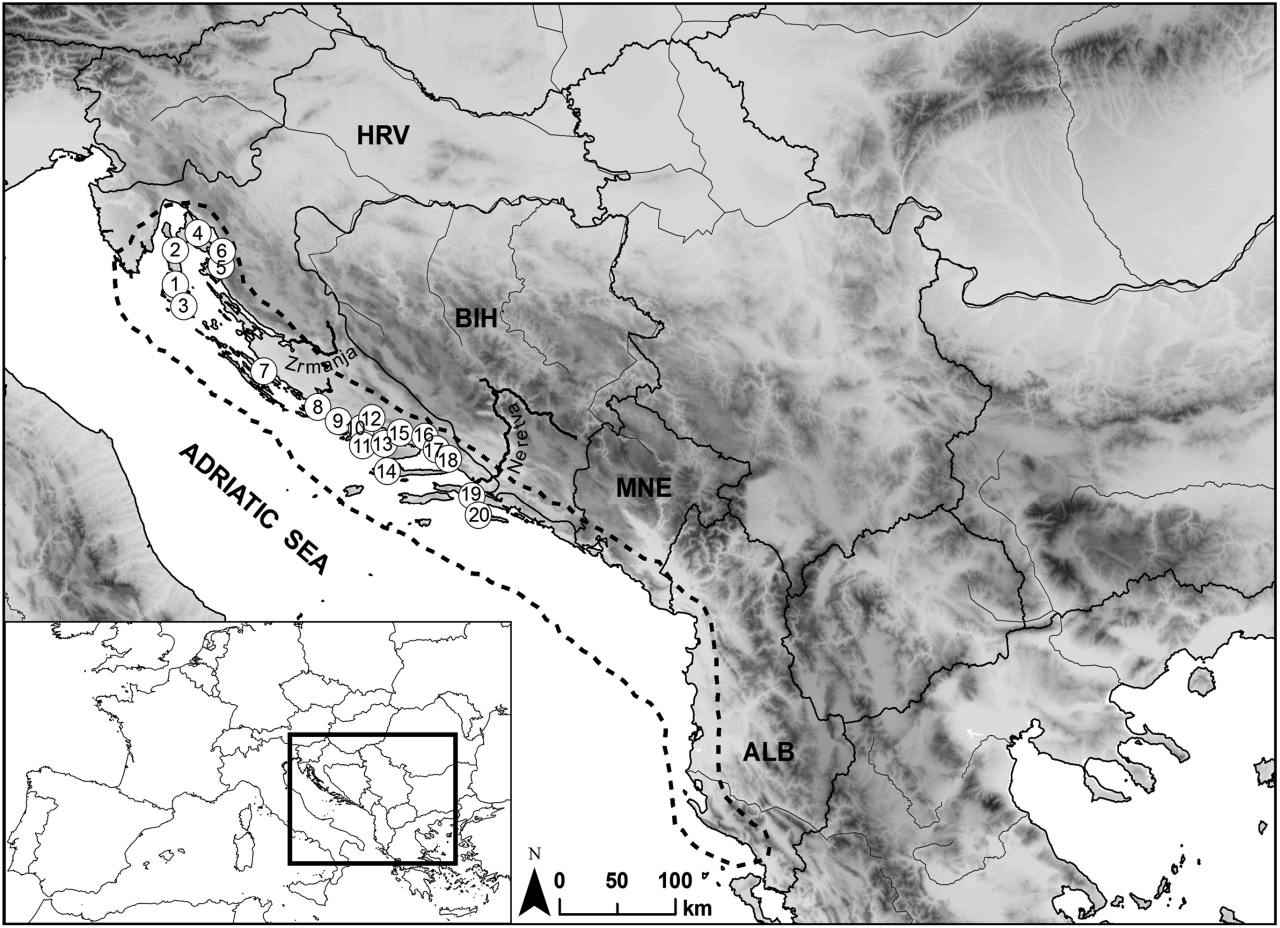
Map of geographic distribution and sampled populations of *Tanacetum cinerariifolium* in Croatia. Dashed line indicates distribution range of *T. cinerariifolium*. Circles numbered 1–20 represent sampled populations.

A total of 411 specimens of *T. cinerariifolium* were sampled from 20 locations along the Adriatic coast in Croatia (Figure 1). Between 13 and 25 samples per location were collected as fresh leaf tissue (Table 1). The vast majority of the species populations is distributed in Croatia, therefore we focused our study exclusively on a wide sampling of Croatian populations.

**Table 1:** Sampling sites and molecular diversity revealed by AFLP markers in 20 Dalmatian pyrethrum populations from Croatia.

No.	AccessionNumber^a^	Population	Latitude (N)^b^	Longitude (E)^b^	Elevation (m)	n	P%	N_pr_	I	H_E_	*DW*
1	MAP02143	Osor	44.70	14.39	2	23	44.18	2	0.242	0.112	56.76
2	MAP02158	Cres	44.96	14.41	72	22	45.70	2	0.246	0.113	58.18
3	MAP02139	Mali Lošinj	44.53	14.47	42	21	50.93	4	0.284	0.131	95.20
4	MAP02156	Krk	45.08	14.67	24	24	52.11	3	0.268	0.118	62.74
5	MAP02148	Gornja Klada	44.82	14.90	589	22	40.64	1	0.214	0.105	48.82
6	MAP02138	Senj	44.93	14.92	6	19	37.61	2	0.214	0.105	42.91
7	MAP02180	Pašman	44.00	15.29	17	22	36.26	3	0.205	0.102	41.45
8	MAP02171	Zlarin	43.70	15.84	45	25	42.33	0	0.234	0.109	45.04
9	MAP02166	Primošten	43.58	16.05	190	22	45.53	4	0.239	0.110	46.09
10	MAP02144	Čiovo	43.51	16.29	143	19	47.22	2	0.251	0.118	48.67
11	MAP02153	Šolta	43.38	16.29	114	19	40.13	0	0.226	0.105	31.59
12	MAP02145	Kozjak	43.58	16.41	516	20	41.15	1	0.220	0.106	31.25
13	MAP02155	Brač	43.38	16.52	116	22	40.81	3	0.216	0.102	47.50
14	MAP02173	Hvar	43.17	16.53	333	21	37.44	0	0.209	0.102	29.40
15	MAP02170	Omiš	43.45	16.70	2	22	38.79	2	0.205	0.102	39.09
16	MAP02142	KotiškiStanovi	43.32	17.06	1335	13	26.14	1	0.160	0.092	18.92
17	MAP02140	Lađena	43.30	17.07	1295	18	34.40	1	0.196	0.099	47.38
18	MAP02141	RavnaVlaška	43.29	17.08	1228	22	34.23	3	0.196	0.096	48.53
19	MAP02184	Pelješac	42.92	17.40	18	14	42.50	1	0.247	0.125	33.18
20	MAP02152	Mljet	42.77	17.45	172	21	33.39	1	0.186	0.094	40.03
		Average					40.57		0.223	0.107	45.64
		Minimum					26.14		0.160	0.092	18.92
		Maximum					52.11		0.284	0.131	95.20

a Accession number from The Collection of Medicinal and Aromatic Plants, Zagreb, Croatia as available at the CPGRD (http://cpgr.zsr.hr);

b N-North; E-East; Coordinates are in degree decimal format; n-sample size; %P-proportion of polymorphic bands; N_pr_ -number of private bands; I-Shannon’s information index; H_E_ -gene diversity of a population assuming Hardy-Weinberg equilibrium; and *DW*-frequency down-weighted marker values.

### DNA isolation and AFLP analysis

The total genomic DNA was isolated from 90 to 110 mg of fresh leaves tissue with DNA-GenElute Plant Genomic DNA Miniprep Kit (Sigma Aldrich, Steinheim, Germany), according to manufacturer’s instructions. Prior to isolation leaves tissue was quickly frozen in liquid nitrogen (−196°C) and ground to a fine powder with TissueLyzer homogenizer set (Qiagene, Hilden, Germany) at 30 Hz for 1 minute. The DNA concentrations were measured using a Qubit Fluorometer (Invitrogen, Carlsbad, California). The AFLP analysis was carried out following the original protocol that was described by Vos et al. [54] with some modifications [55]. Restriction of the genomic DNA was performed using the restriction enzymes *Eco*RI and *Mse*I. Specific adapters were ligated using T4 DNA Ligase (Fermentas, Thermo Scientific). The restriction digestion and adapter ligation, pre-amplification and selective amplifications were performed in a GeneAmp PCR System 9600 (Applied Biosystems, Carlsbad, SAD). Six primer combinations were selected for amplification; VIC-*Eco*RI-ACGC+*Mse*I-AGA, NED-*Eco*RI-AGA+*Mse*I-CAG, VIC-*Eco*RI-ACG+*Mse*I-CGA, FAM-*Eco*RI-ACC+*Mse*I-CGA, NED-*Eco*RI-AGA+*Mse*I-CGA, PET-*Eco*RI-ATG+*Mse*I-CGA. The amplified fragments were separated by capillary electrophoresis in an ABI3130xl Genetic Analyzer (Applied Biosystems, Carlsbad, USA). The amplified DNA fragments in the size range of 50 to 125 bp were scored with GeneMapper 4.0 software (Applied Biosystems, Carlsbad, USA) as being present (1) or absent (0) in order to create a binary matrix.

### Data analysis

The selection of markers for further statistical analysis and the estimation of the error rate per primer combination were performed as suggested by Herrmann et al. [56]. Thirteen DNA samples were used as duplicated samples, two of which were chosen for multiple controls, while 14 samples represented negative controls. The output from GeneMapper (fragment size and peak heights) was imported into the marker selection algorithm scanAFLP 1.3r (www-leca.ujf-grenoble.fr/logiciels.htm).

The genetic diversity within populations was estimated by determining the percentage of polymorphic bands *(%P)*, the number of private alleles *(N_pr_)* and Shannon’s information index (*I*) [57]–[58]. Shannon’s information index was used to measure the total diversity (*H_T_*) as well as the average intra-population diversity (*H_P_*) and the diversity within (*H_P_/H_T_*) and among populations [*(H_T_–H_P_)/H_T_*]. As a measure of divergence, the frequency down-weighted marker values (*DW*) [23] were calculated using the software AFLPdat [59].

The marker allelic frequencies were estimated from the observed frequencies of the fragments using a Bayesian method with non-uniform prior distribution of allele frequencies proposed by Zhivotovsky [60] as implemented in AFLP-Surv ver. 1.0 [61]. This approach assumes Hardy-Weinberg equilibrium (*F_IS_* = 0) justified by the outcrossing nature of Dalmatian pyrethrum. The obtained allelic frequencies were used in the analysis of genetic diversity within and between populations following the treatment of Lynch and Milligan [62]. The population genetic structure was described in terms of the total gene diversity (*H_T_*), the average gene diversity within populations (*H_W_*), the average gene diversity among populations in excess of that which was observed within populations (*H_B_*), and Wright’s *F_ST_* statistics. The expected heterozygosity or Nei’s gene diversity (*H_E_*) [63] was calculated for each population as well as pairwise Nei’s genetic distances (*D_NEI72_*) [62], [64]. The unrooted tree was constructed using the Fitch-Margoliash method in the PHYLIP ver. 3.6b software package [65]. A total of 1,000 distance matrices were computed by bootstrapping using the program AFLP-Surv, and the bootstrap values were calculated from FITCH and CONSENSE within PHYLIP.

An analysis of molecular variance (AMOVA) [66] was used to partition the total genetic variance within and among populations using Arlequin ver. 2.000 [67]. The variance components were tested statistically by non-parametric randomization tests using 10,000 permutations. Pairwise population comparisons that were examined with AMOVA resulted in *φ*_ST_ values that were equivalent to the proportion of the total variance that was partitioned between the two populations and could be interpreted as the inter-population distance average between any two populations [68].

The genetic structure was assessed using the model-based clustering approach implemented by the software STRUCTURE 2.3.3 [69], which simultaneously identifies clusters and assigns individuals to clusters using a Bayesian approach. Ten runs of STRUCTURE were performed by setting the number of clusters from 1 to 21 (*K* = 1–21). Each run consisted of a burn-in period of 200,000 steps followed by 10^6^ MCMC (Monte Carlo Markov Chain) replicates assuming an admixture model and correlated allele frequencies. No prior information was used to define the clusters. The calculations were carried out at the Bioportal of the University of Oslo (http://www.bioportal.uio.no/). The most likely number of clusters (*K*) was selected by calculating an *ad hoc* statistic *ΔK* based on the rate of change in the log probability of the data between successive *K* values as described by Evanno et al. [70] and implemented in Structure-sum-2011 [71]. The runs with the maximum likelihood were selected, and by averaging the estimated membership coefficients of the individuals, the proportion of ancestry of each population in each of the clusters was calculated.

A further population mixture analysis was conducted using BAPS ver. 5.3 [72] (a) without using the geographic origin of the samples as an informative prior (*‘clustering of individuals’*) and (b) with this prior (*‘spatial clustering of individuals’)* [73]. BAPS was run with the maximal number of clusters (K) set to 20, and each run was replicated 10 times. The results of the mixture analysis were used as input for population admixture analysis [74] with the default settings to detect admixture between the clusters.

Isolation by distance (IBD) among the populations was tested using the method of Rousset [75]. Mantel’s test [76] was used to compute and test the linear correlation between the matrix of the natural logarithm of geographic distances (in km) between pairs of populations and the matrix of pairwise *F_ST_*/(1-*F_ST_*) ratios.

The plausibility of patterns of isolation by environmental distance (IBED) was evaluated, where differences in the bioclimatic variables among the sampling sites would better explain the genetic distance patterns [33], [77]. The climate data for 25 sampling sites were obtained from the WorldClim database at a spatial resolution of approximately 1 km^2^ [78]. The ecological characteristics of the sampling sites were described using 19 bioclimatic variables (11 temperature- and 8 precipitation-related) representing the annual trends, seasonal variations and extremes in temperature and precipitation (Table 2). Bioclimatic variables were used for the Principal Component Analysis (PCA) and subsequently to calculate the environmental distances among populations based on principal component scores rather than original values to avoid intercorrelation among variables. PCA was performed using the PROC PRINCOMP procedure in SAS. The number of principal components was determined by checking the eigenvalues of the principal components and the cumulative proportion of the explained variance. The biplot was constructed using two principal components showing populations and bioclimatic variables (as vectors). The standardized scores of the first three principal components (eigenvalue >1) were multiplied by the root of their eigenvalues, and the pairwise Euclidean distance between populations were calculated to obtain an environmental distance matrix.

**Table 2:** Correlations between 19 environmental variables (BIO01–BIO19) and the first three principal components.

No.	Environmental variable	PC1		PC2		PC3	
BIO01	Annual Mean Temperature	0.942	***	0.244	^ns^	0.095	^ns^
BIO02	Mean Diurnal Range (Mean of monthly (max temp - min temp)	0.678	**	0.546	*	−0.357	^ns^
BIO03	Isothermality (P2/P7) (*100)	0.733	***	0.474	*	−0.251	^ns^
BIO04	Temperature Seasonality (standard deviation *100)	−0.346	^ns^	0.545	*	−0.497	*
BIO05	Max Temperature of Warmest Month	0.935	***	0.322	^ns^	0.027	^ns^
BIO06	Min Temperature of Coldest Month	0.928	***	0.138	^ns^	0.218	^ns^
BIO07	Temperature Annual Range (P5–P6)	0.518	*	0.652	**	−0.482	*
BIO08	Mean Temperature of Wettest Quarter	0.866	***	0.285	^ns^	−0.179	^ns^
BIO09	Mean Temperature of Driest Quarter	0.935	***	0.270	^ns^	0.078	^ns^
BIO10	Mean Temperature of Warmest Quarter	0.938	***	0.263	^ns^	0.082	^ns^
BIO11	Mean Temperature of Coldest Quarter	0.950	***	0.199	^ns^	0.131	^ns^
BIO12	Annual Precipitation	−0.712	***	0.653	**	0.234	^ns^
BIO13	Precipitation of Wettest Month	−0.539	*	0.747	***	0.380	^ns^
BIO14	Precipitation of Driest Month	−0.860	***	0.363	^ns^	−0.275	^ns^
BIO15	Precipitation Seasonality (Coefficient of Variation)	0.795	***	0.061	^ns^	0.476	*
BIO16	Precipitation of Wettest Quarter	−0.503	*	0.789	***	0.348	^ns^
BIO17	Precipitation of Driest Quarter	−0.844	***	0.425	^ns^	−0.203	^ns^
BIO18	Precipitation of Warmest Quarter	−0.873	***	0.380	^ns^	−0.196	^ns^
BIO19	Precipitation of Coldest Quarter	−0.492	*	0.489	*	0.695	***
	Eigenvalue	11.55		4.00		1.97	
	% variance	60.79		21.04		10.39	

ns - non-significant;

*significant at P<0.05;

**significant at P<0.01;

***significant at P<0.001.

A simple Mantel’s test was used to test the linear correlation between the matrix of environmental and genetic [*F_ST_*/(1-*F_ST_*)] distances, and a three-way Mantel’s test was conducted between the same distances matrices while considering the geographical distances among populations. Thus, the correlations between the residual environmental and genetic distances among the analyzed populations represent isolation by environmental distance (IBED) pattern. The significance levels were obtained after 10,000 permutations as implemented in NTSYS-pc ver. 2.10s [79].

## Results

### ScanAFLP

The initial number of polymorphic bands that were obtained with six selective primer combinations equaled 850, while after the applying scanAFLP, the remaining dataset consisted of 593 polymorphic markers (43.35% of the initial number of polymorphic markers). The estimated error rate per primer combination ranged from 0.68% to 3.50% with a mean of 1.83%, which is within the typical range found in AFLP datasets [80].

### Within population genetic diversity

**Figure 2:**
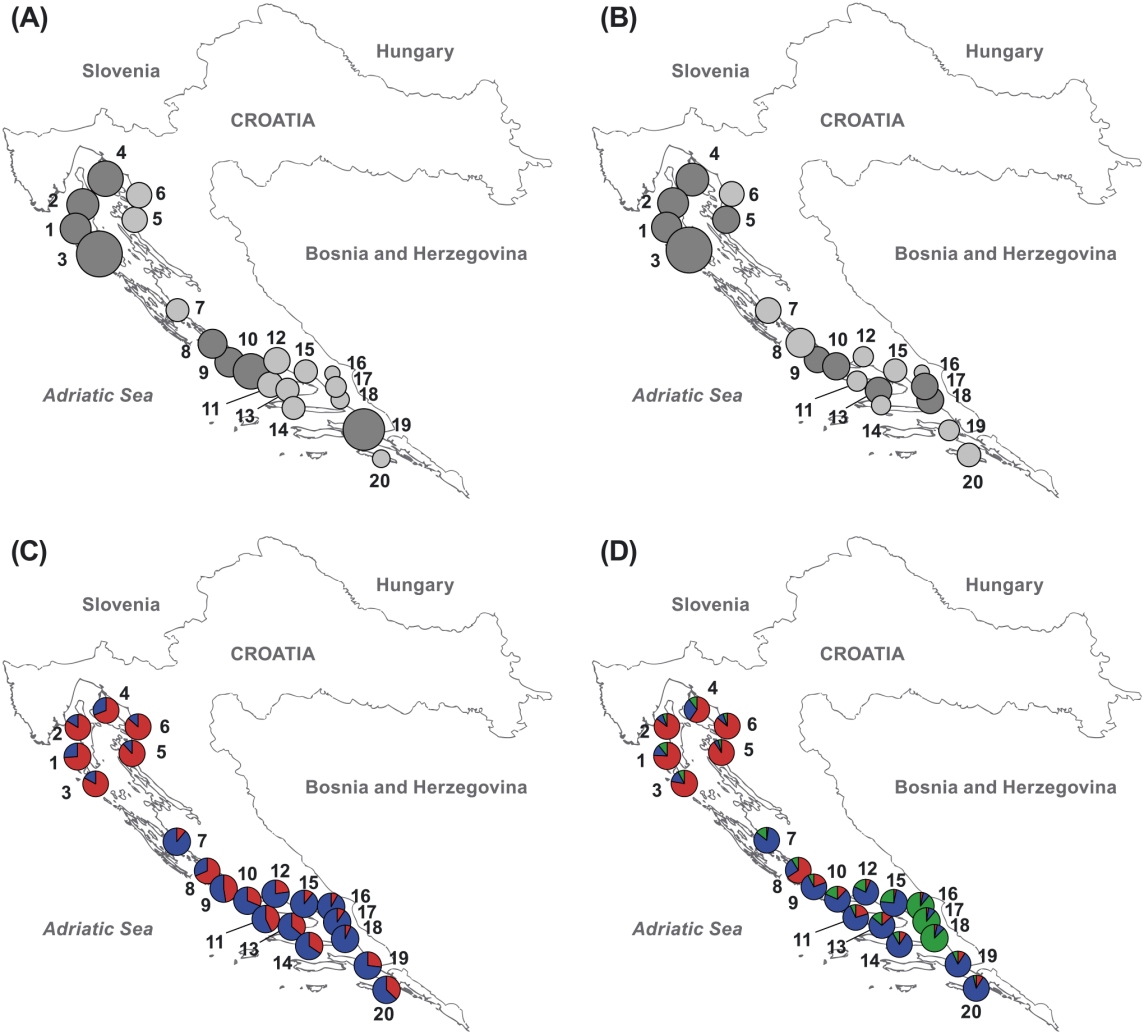
AFLP variation of *Tanacetum cinerariifolium*. A) Gene diversity (*H_E_*), B) Frequency down-weighted marker values (*DW*). In (A) and (B), the size of the circles is proportional to the depicted values (dark gray color represents values above average across populations and light gray represents values below average). C) Bayesian analysis of the population structure using the software STRUCTURE assuming *K* = 2, D) Bayesian analysis of the population structure using the software STRUCTURE assuming *K* = 3. In (C) and (D), the proportions of the ancestry of each population in each of the defined gene pools are color-coded (gene pool A red; gene pool B-blue; and gene pool C-green).

The percentage of polymorphic markers varied among the populations with the highest proportion in population 4 (52.11%) and the lowest in population 16 (26.14%). Shannon’s information index indicated that the total diversity (*H_T_*) was 0.297, while the average intrapopulation diversity (*H_P_*) was 0.223. The proportion of diversity within populations (75.00%) was considerably higher than the proportion of diversity among populations. Out of 593 scored markers, 36 were private. The populations with the most private alleles (4) were pops. 3 and 9, while no such alleles were found in the pops. 8, 11 and 14. The population 3 had the highest level of expected heterozygosity (*H_E_*), and the population 16 had the lowest (0.131 and 0.92, respectively) (Table 1, Figure 2A). The frequency down-weighted marker values (*DW*) ranged from 18.92 in the population 16 to 95.20 in the population 3 (Table 1, Figure 2B).

### Genetic structure among populations

A moderate level of population differentiation was observed (*F_ST_* = 0.078) ranging from 0.018 between pops. 10 and 11 to 0.161 between 6 and 18.

The AMOVA analysis showed that although the most of the genetic diversity was attributable to differences among individuals within populations (85.78%), the *φ*_ST_ values among populations were highly significant (*φ*_ST_ = 0.141 *p<*0.0001) confirming the existence of population differentiation. The *φ*_ST_ values ranged from 0.049 between pops. 4 and 11 to 0.278 between pops. 5 and 16.

**Figure 3:**
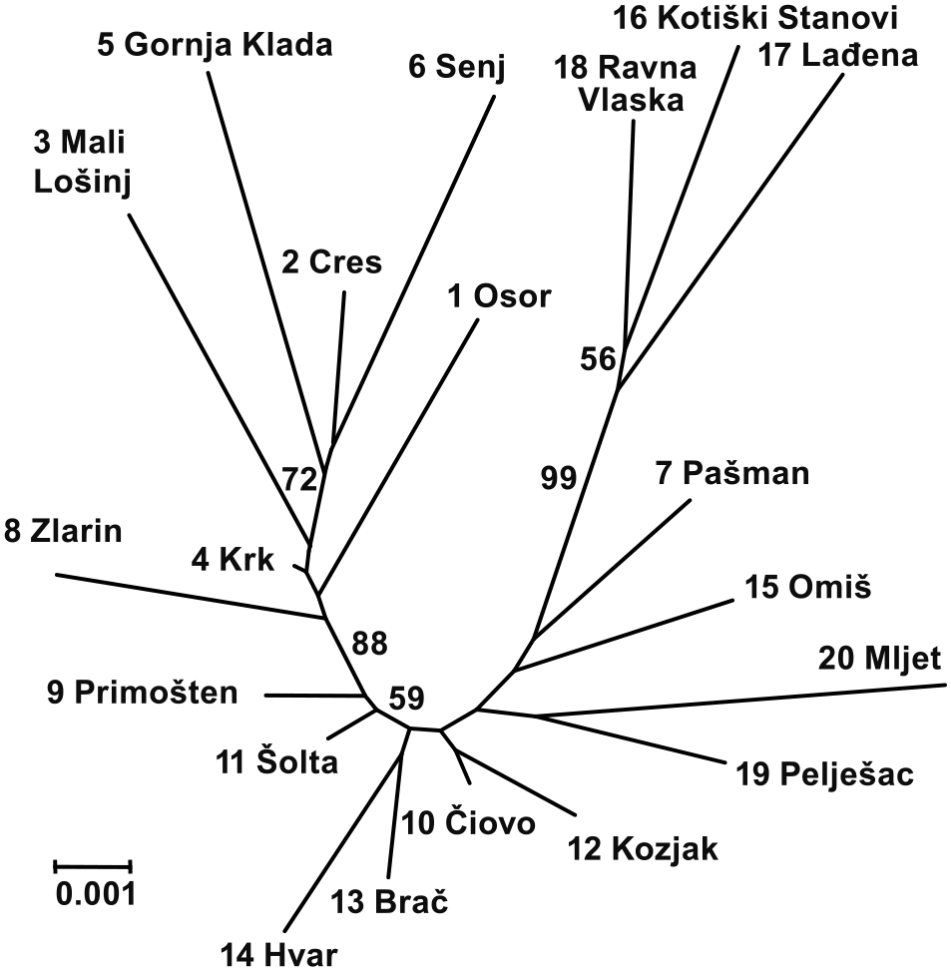
Unrooted Fitch-Margoliash tree of 20 *Tanacetum cinerariifolium* populations. Bootstrap values greater than 50% that were obtained after 1000 permutations are indicated on the branches.

Nei’s distance (*D_NEI72_*) varied from 0.002 between pops. 10 and 11 to 0.022 between pops. 6 and 18 with an average value of 0.010. The pattern of grouping in the Fitch-Margoliash tree suggests a direct relationship between the populations and their geographical origins. This pattern revealed the existence of two clades (northern vs. middle and southern Adriatics) supported by a bootstrap value of 88%. Inside the northern clade, the inland pops. 5 and 6 along with the population 2 separated from the northern island populations with a bootstrap value of 72%. The pops. 9 and 11 separated from pops. 8 and 4 with a bootstrap value of 88%. The populations from Mt. Biokovo (pops. 16, 17 and 18) grouped together and showed a clear separation from the remaining southern populations with a high bootstrap support of 99% (Figure 3).

The results of the STRUCTURE analysis are reported in Figure 2C and 2D. The average estimates of the likelihood of the data, conditional on a given number of clusters, *[ln P(X|K)],* were obtained for each of the 10 independent runs for each *K* (from *K* = 1 to 21). The estimates of *[ln P(X|K]* increased progressively as *K* increased. Using these estimates, the highest *ΔK* value was observed for *K* = 2 (302.59). The second highest *ΔK* value was observed for *K* = 3 (180.78). At *Ks* values greater than three, the average *[ln P(X|K)]* values decreased, while the variances increased substantially and, therefore, the *ΔK* values were considerably lower (0.10 to 5.80) in comparison to *K* = 2 and 3. Thus, the hypothesis of having more than three clusters (gene pools) was far from optimal.

The proportions of membership (ancestry) of each individual in each of the gene pools were calculated for *K* = 2 to 3 based on the run with the highest *[ln P(X|K)]*, and populations were assigned to a particular gene pool (A, B or C). The results were in accordance with the results that were obtained using the distance-based method. Representative populations of each gene pool that were identified in the STRUCTURE analysis were located in proximity of each other. At *K* = 2, the representative populations of gene pool A were mainly those from the northern Adriatic, while the populations from the middle and southern Adriatic were representative of a separate gene pool B. The most admixed populations were 9 and 11 with almost equal proportions of ancestry from the two gene pools (Figure 2C). At *K* = 3, a newly formed gene pool C was identified and was predominant in the pops. 16, 17 and 18 from Mt. Biokovo (Figure 2D).

The BAPS mixture with or without spatially informative priors resulted in the congruent assignment of individuals to two clusters. The best partitions received log marginal likelihoods of −51646.2626 at P = 1 (without using geographical coordinates as informative priors) and −51793.7943 at P = 1 (spatial clustering). The results were consistent with the results of the STRUCTURE analysis (Figure S1).

### Isolation by distance (IBD) and isolation by environmental distance (IBED)

**Figure 4:**
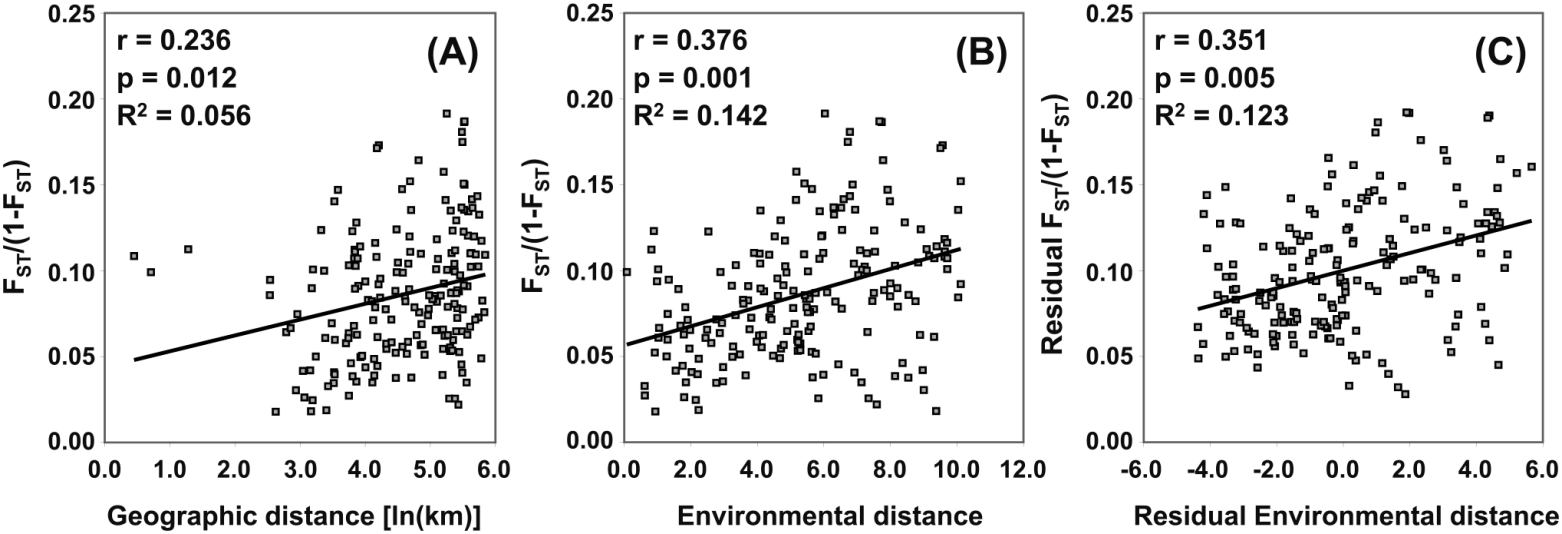
Isolation by distance and isolation by environmental distance. Plots of Mantel’s test showing the relationships between A) the geographical and genetic distances, B) the environmental and genetic distances and C) the residual environmental and genetic distances considering the geographical distances between 20 Dalmatian pyrethrum populations.

A significant correlation was found between the genetic and geographical distances (*r* = 0.236; *P_Mantel_* = 0.012), suggesting that 5.6% of the genetic differentiation between populations could be explained by geographical distances (Figure 4A).

As expected, 19 environmental variables were highly intercorrelated. Out of 171 pairwise correlations, 107 (62.57%) were significant at P<0.05 (Table S1). In 49 cases the strong positive correlations (*r*>0.70) were detected, mostly among eleven temperature-related variables (BIO01–BIO11) and among eight precipitation-related variables (BIO12–BIO19). The strong negative correlations (*r*<−0.70) were found in 10 cases, usually between temperature-related and precipitation-related variables.

**Figure 5:**
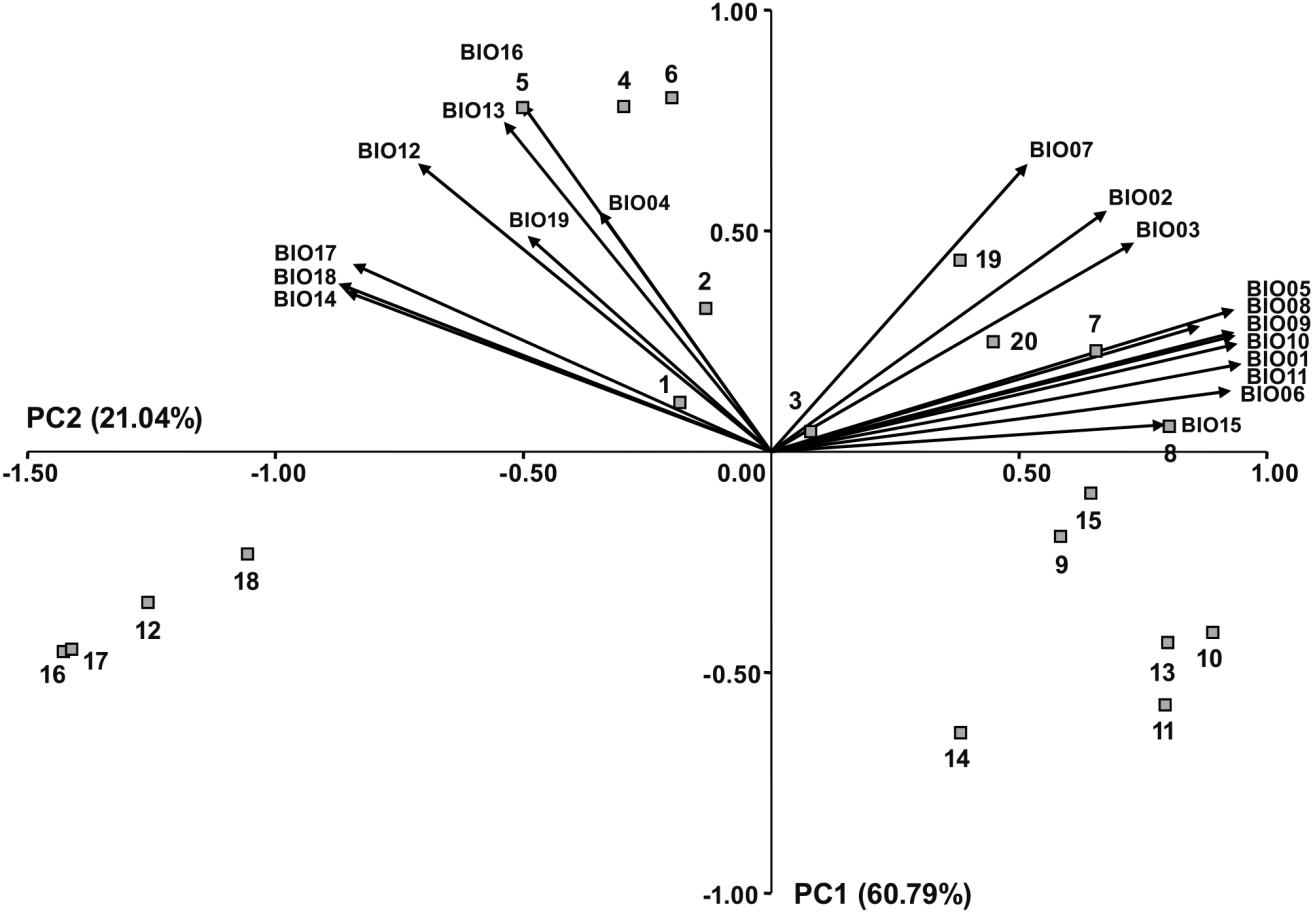
PCA plot based on 19 environmental variables (BIO01–BIO19) describing 20 *Tanacetum cinerariifolium* sampling sites.

A Principal Component Analysis based on the correlation matrix revealed that first three principal components had an eigenvalue >1 and explained more than 90% of the total variation (Table 2). Eight temperature-related variables (BIO01, BIO03, BIO05, BIO06, BIO08, BIO09, BIO10 and BIO11) and one precipitation-related variable (BIO15) were highly positively correlated (r>0.70; P<0.001) with the first principal component (PC1), while four precipitation-related variables (BIO12, BIO14, BIO17 and BIO18) were highly negatively correlated (r<−0.70; P<0.001). Strong positive correlations (r>0.70; P<0.001) were observed between the second principal component (PC2) and two precipitation-related variables (BIO13 and BIO16). The biplot that was constructed by the two principal components showing populations and 19 environmental factors (as vectors) is presented in Figure 5. PC1, explaining 60.79% of the total variation, separated the middle (pops. 7–11) and southern (pops. 13–15, 19 and 20) Adriatic populations occupying warmer habitats from pops. 12, 16, 17 and 18, which were sampled at higher elevation (516 to 1,335 m.a.s.l.). Along PC2, explaining 21.04% of the total variation, the northern Adriatic populations (pops. 1–6), occupying habitats that were characterized by greater rainfall, were separated from the remaining populations. The average Euclidean distance between the pairs of populations that was calculated based on the standardized scores of the first three principal components was 5.34, ranging from 0.08 (between pops. 16 and 17) to 10.12 (between pops. 8 and 16, respectively).

A greater correlation was found between the genetic and environmental distances (*r* = 0.376; *P_Mantel_*<0.001) than between the genetic and geographical distances (Figure 4B). After considering the effect of the geographical distances in a three-way Mantel test, the correlation between the residual genetic and residual environmental distances remained significant (*r* = 0.351; *P_Mantel_* = 0.005) suggesting that isolation by environmental distance (IBED) explains 12.3% of the observed genetic pattern (Figure 4C). The removal of the effect of environmental variation resulted in a still significant but much lower correlation between the genetic and geographic distances (*r* = 0.189; *P_Mantel_* = 0.039).

## Discussion

Identification of genetic diversity patterns is of fundamental importance for effective management and species conservation [7], and as we expand our knowledge about the genetic diversity and structure of natural populations, it subsequently becomes important to understand the mechanisms behind the determined patterns. Therefore, the genetic diversity and population genetic structure of Dalmatian pyrethrum must be analyzed both in the context of demographic history and biological traits of the species [3]. Additionally, understanding the impact of geographical distances and consequently restricted gene flow among the analyzed populations as well as climatic characteristics of the sampling sites can contribute to a better comprehension of the relationships between the populations. Furthermore, the fact that human historical activities might have influenced the observed pattern must not be excluded.

The results of our study show genetic differentiation of *T. cinerariifolium* into two (Figure 2C) to three (Figure 2D) distinct groups. At *K = *2 it is plausible that the north-south split in the study area is due to widely spaced canyon of karstic river Zrmanja in northern Dalmatia as karst rivers are often major barriers to the otherwise continuous distribution of species. The obtained results are somewhat similar to the phylogeographical patterns detected in *Edraianthus tenuifolius* [10], *Campanula pyramidalis* [25] and *Cardamine maritima* [81] showing either phylogeographical or taxonomical split in the area of river Neretva valley (central Dalmatia). However, the fact that north-south split might be the artefact of genetic impoverishment (discussed further in the text) of central and southern population must not be neglected. With additional splitting of southern gene pool at *K = 3*, a new pattern that is congruent with the survival of the species in multiple refugial areas, followed by a period (s) of isolation occurs. Three genetically and geographically distinct and well-defined groups of Dalmatian pyrethrum populations therefore support the “refugia within refugia” model that was already confirmed in numerous studies in the Balkan Peninsula [10], [21], [23], [24]. The restricted gene flow among inter-refugial populations has led to genetic differentiation, resulting in well-defined gene pools [82]. Among the 20 populations that were included in this study, the highest levels of gene diversity, private alleles and *DW* were found in group of northern Adriatic populations from Kvarner bay. The genetic structure of these populations, which are grouped together within the same cluster as revealed by the STRUCTURE analysis, suggests their ancient origin and significant isolation through a recent period of evolution [17] Such northerly located microrefugia for a thermophytic species like Dalmatian pyrethrum is quite unexpected but is strongly supported by the obtained results. Previous research in this region on Dalmatian wall lizard (*Podarcis melisellensis)* [21], nose-horned viper (*Vipera ammodytes)* [83] and *Edraianthus tenuifolius* [10] found no evidence of a refugium that far north. Our results could possibly be explained by numerous glaciations and interglaciation periods that are characteristic of the Quaternary and that were accompanied by vast species migrations. During the last interglacial period (approximately 130,000 yr. B. P. to 116,000 yr. B. P.), the global climate was warmer than at present [84]–[85], and it is possible that the populations that were initially located in the more southern and thus warmer parts of Adriatic basin expanded far to the north-west. By tracking suitable microhabitats some populations survived during the cold period, and isolation from their pool of origin eventually has led to strong genetic differentiation that can now be detected through northern Adriatic gene pool. These microhabitats in Kvarner bay served as microrefugia, and at the end of the last glacial period, these protected and long-lived populations recolonized the northern Adriatic regions. However, a major flaw of this hypothesis is that in this scenario, there should be at least some evidence of ancient populations in the southern parts of the Adriatic basin. Generally, in more southern populations, the levels of gene diversity, number of private alleles and *DW* decrease. Overall, the detected pattern is not in concordance with the general predictions that the highest levels of diversity can be detected among southern regions of species distribution range [5], [82], thus suggesting either a more recent origin of these populations or some past event which resulted in abrupt changes of their genetic structure. At this point, human activity and the excessive gathering of this species during the last century, primarily in the southern and, to a lesser extent, in the northern parts of Adriatic, must be considered. In fact, until the 1850s, the only reliable source of pyrethrum material was from wild plants [45]; hence, it is very likely that overexploitation alone is responsible for such low gene diversity and *DW*. An exception is the population 19 located in the southern parts of the species’ range. Because it exhibits high levels of gene diversity accompanied by low levels of private alleles and *DW*, this population is likely the zone of secondary contact between the divergent lineages and is anthropogenic in origin. Additionally, some populations show a quite admixed pattern comprising large fractions of different gene pools (pops. 8, 9, 11, etc.). The specific structure of these populations could also easily be a consequence of the admixture of divergent lineages through secondary contact after postglacial colonization, however, more likely explanation of the obtained results could lie in the cultivation practices of Dalmatian pyrethrum, which began in 1850s near Dubrovnik (Southern Dalmatia) and rapidly expanded along the Dalmatian coastal region and the islands [45]. These populations could have been founded through human activity by the exchange of seed material between farmers from different geographical regions. It is well documented that anthropogenic activities foster genetic homogenization and decrease genetic differentiation among populations [36], [86]–[87]. Therefore, we hypnotize that for southern populations overexploitation and process of cultivation are responsible for substantial loss of private alleles, levels of gene diversity and *DW*. Moreover, extensive seed exchange between different populations caused admixture as observed in pops. 8, 9 and 11. Thus, this phylogeographical study could represent typical example of humans’ shaping the genetic structure of investigated plant species. The genetic impoverishment as a result of either overexploitation of wild resources or cultivation practices has also been demonstrated for American ginseng (*Panax quinquefolius* L.) [35], *Rheum tanguticum* [88], *Scutellaria baicalensis* [37], *Eucommia ulmoides* [89], *Moringa peregrina* [90], etc.

The lowest values of gene diversity were detected in the populations from Mt. Biokovo (pops. 16, 17 and 18) (*H_E_* = 0.092–0.096), which were also identified as a separate gene pool by the Bayesian cluster analysis. The Mt. Biokovo populations (pops. 16, 17 and 18) were sampled in geographically and ecologically marginal areas of the species distribution, and it is generally expected that in such peripheral populations, the amounts of diversity may be reduced [91]. This reduction can be the result of, small population size, increased isolation and consequently reduced gene flow [92]–[93]. At such high elevation (greater than 1200 m.a.s.l.) with atypical climatic conditions for the species, it is highly unlikely that the spontaneous formation and development of populations of such a pronouncedly thermophytic species could be expected in the present day. However, during some past periods that were warmer than present (Holocene Climatic Optimum) [94], such altitudinal shifts in species distribution are quite expected and have already been documented for the western Balkans [10]. Therefore, these populations can be treated as relict populations, which are ecologically isolated from other populations. Evidently, a sufficient period of time has passed for this group of populations to diverge and form a separate, well-defined gene pool.

Evidence that both geography and environmental heterogeneity contributed significantly to the obtained patterns of genetic divergence was found. The IBD analysis showed positive and significant correlations, which could in principal be the result of successive postglacial colonization from the refugia [28]. However, a large part of unexplained genetic variations among the populations still remains after accounting for the geographical distances. Furthermore, a significant correlation between environmental and genetic variation was found, and 12.3% of the genetic variation was explained by environmental heterogeneity. The populations from Mt. Biokovo (pops. 16, 17 and 18), located at significantly higher elevation (from 1295 to 1335 m.a.s.l.), inhabiting environmentally distinct habitat in comparison to the remaining populations, are typical cases of environmental isolation. It is well acknowledged that microclimate strongly influences plant phenology [95]–[96] whereas differences in temperature, photoperiod and moisture are major factors in shaping patterns of flowering and fruiting [97]. Due to cooler temperatures and higher precipitation the populations from Mt. Biokovo tend to flower later (cca. 20 days) than do the low-elevation populations (authors observations); therefore, there is no phenological overlap between them, resulting in genetic isolation and differentiation of Mt. Biokovo populations (pops. 16, 17, 18). In addition, great influence of sea surface on coastal area ecological conditions and consequently on vegetation cover [25] could have also served as a stimulus for such strong differentiation between high altitude and lowland populations. Bathymetric configuration of Adriatic Sea floor is characterized by very deep valley with steep slopes in southern region (South Adriatic Basin) and relatively shallow and plain central and northern region [98]–[99]. For that reason northern Adriatic part has experienced more pronounced substantial shifts in shoreline during the sea-level fluctuations, while the southern regions remained almost unchanged [100]. Therefore, in the past when the sea level was significantly lower than today [99], [101], populations from middle and especially southern parts of Dalmatia were located in proximity of the sea as they are today. However, this is not the case for high altitude areas as they were never located near sea surface, which might have resulted in divergent evolution particularly observed in Mt. Biokovo populations (pops. 16, 17 and 18). The strong influence of environmental factors on genetic divergence of populations has already been documented for several plant [77], [102] and animal species [31], [33]. For example, Temunović et al. [77] determined genetic structuring of Croatian Continental and Mediterranean ash trees populations (*Fraxinus angustifolia* Vahl) due to contrasting ecological conditions of the sampling sites. Similarly, strong fine-scale spatial genetic structure associated with habitat factors was detected in continuous population of *Biscutella laevigata* [102]. For the Franciscana dolphins genetically isolated populations in environmentally distinct areas of Argentina were observed [33] and for the grey wolf (*Canis lupus*) genetic differentiation among local populations was correlated to contrasting climatic conditions [31].

In our study the detected IBED pattern explained more than twice the genetic divergence than did the IBD pattern (12.3 vs. 5.6%, respectively). Therefore it is clear that the revealed genetic differentiation in Dalmatian pyrethrum is more strongly influenced by the bioclimatic conditions of the sampling sites rather than by geographic distances *per se*.

## Conclusion

The aim of this study was to analyze the genetic diversity and structure of natural Dalmatian pyrethrum (*Tanacetum cinerariifolium* Trevir. /Sch./ Bip.) populations from Croatia. Overall, our results show that, as a thermophytic plant, Dalmatian pyrethrum could have survived the Quaternary climatic oscillation along the eastern Adriatic coast by tracking suitable habitats, i.e., microrefugia. Northern Adriatic populations, which were identified as a separate cluster by the STRUCTURE analysis, are characterized by high levels of gene diversity and *DW* and should be treated as conservation priority sites. Human influence through overexploitation and cultivation has resulted in the low genetic variability of southern Adriatic populations. The results also provide evidence that both IBD and especially IBED contributed to the genetic differentiation among some populations.

## Supporting Information

Figure S1 **Genetic structure of** ***T. cinerariifolium*** **populations.** (A) using software Structure and assuming *K* = 2, (B) using Structure and assuming *K* = 3; (C) using BAPS without spatially informative prior, and (D) using BAPS with spatially informative prior. Each individual plant is represented by a single vertical line divided into colors. Each color represents one cluster, and the length of the colored segment shows the individual’s estimated proportion of membership in that cluster. White lines separate populations that are labelled below the figure (1–20).
(TIF)

Table S1 **Correlations coefficients (r; lower diagonal) and its significance (P-values; upper diagonal) among 19 environmental variables at 20 sampling sites of** ***T. cinerariifolium*** **in Croatia.**
(DOCX)
