# Supplementary figures and images for: Genetic Diversity and Structure of Dalmatian Pyrethrum (Tanacetum cinerariifolium Trevir. /Sch./ Bip., Asteraceae) within the Balkan Refugium
Source: PLoS One. 2014 Aug 14;9(8):e105265. doi: 10.1371/journal.pone.0105265 (PMC4133326; doi:10.1371/journal.pone.0105265)

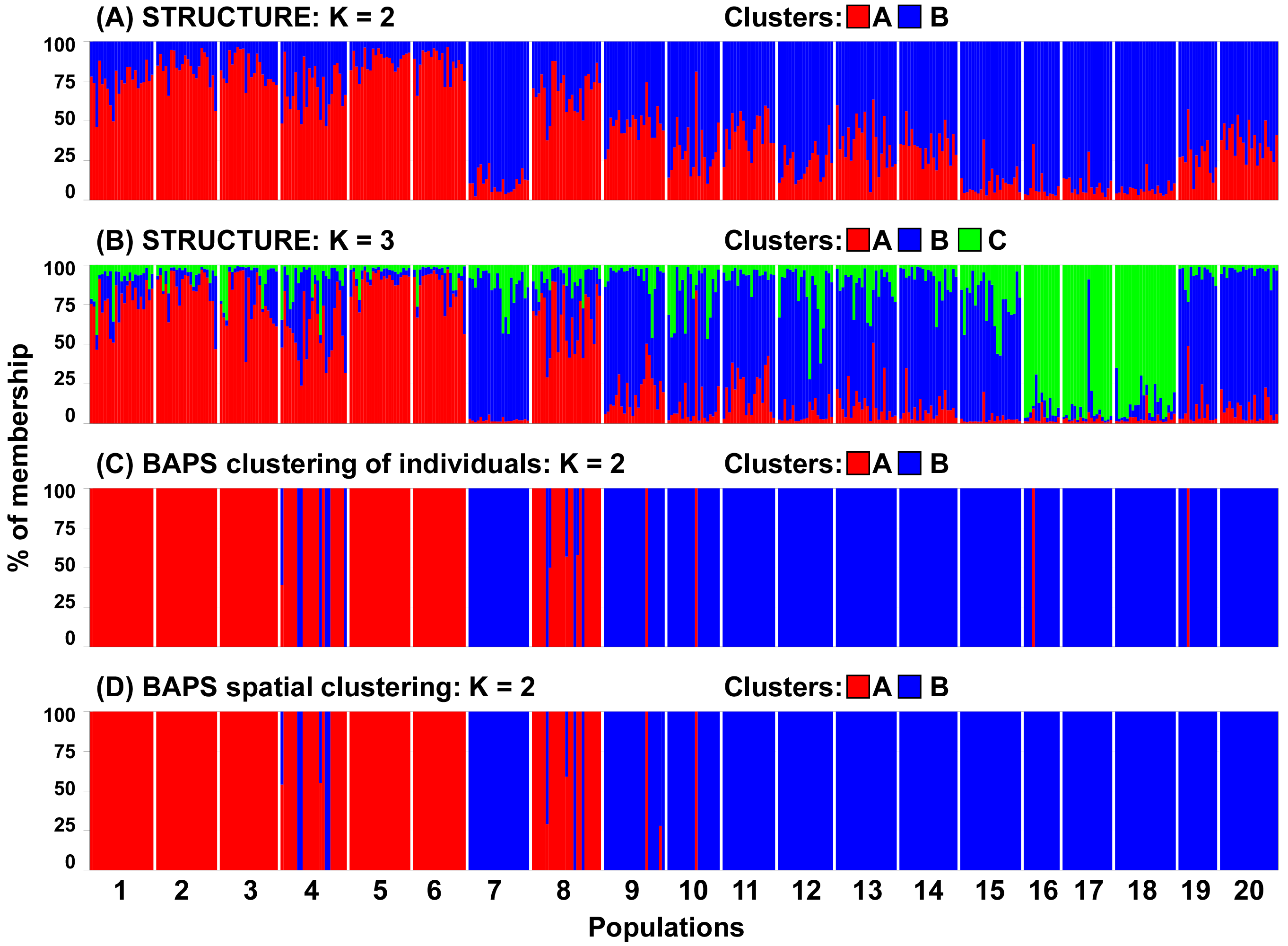

Supplement: Figure S1 — Genetic structure of T. cinerariifolium populations. (A) using software Structure and assuming K = 2, (B) using Structure and assuming K = 3; (C) using BAPS without spatially informative prior, and (D) using BAPS with spatially informative prior. Each individual plant is represented by a single vertical line divided into colors. Each color represents one cluster, and the length of the colored segment shows the individual’s estimated proportion of membership in that cluster. White lines separate populations that are labelled below the figure (1–20). (TIF) [file pone.0105265.s001.tif]
