# Supplementary material for: Genetic Diversity and Structure of Dalmatian Pyrethrum (Tanacetum cinerariifolium Trevir. /Sch./ Bip., Asteraceae) within the Balkan Refugium
Source: PLoS One. 2014 Aug 14;9(8):e105265. doi: 10.1371/journal.pone.0105265 (PMC4133326; doi:10.1371/journal.pone.0105265)
Supplement: Table S1 — Correlations coefficients (r; lower diagonal) and its significance (P-values; upper diagonal) among 19 environmental variables at 20 sampling sites of T. cinerariifolium in Croatia. (DOCX) [file pone.0105265.s002.docx]

Supporting Table 1. Correlations coefficients (r; lower diagonal) and its significance (P-values; upper diagonal) among 19 environmental variables at 20 sampling sites of *T. cinerariifolium* in Croatia

| No. | Environmental variable | Environmental variable | | | | | | | | | | | | | | | | | | |
| --- | --- | --- | --- | --- | --- | --- | --- | --- | --- | --- | --- | --- | --- | --- | --- | --- | --- | --- | --- | --- |
|  |  | BIO01 | BIO02 | BIO03 | BIO04 | BIO05 | BIO06 | BIO07 | BIO08 | BIO09 | BIO10 | BIO11 | BIO12 | BIO13 | BIO14 | BIO15 | BIO16 | BIO17 | BIO18 | BIO19 |
| BIO01 | Annual Mean Temperature |  | 0.001 | 0.001 | 0.337 | 0.000 | 0.000 | 0.012 | 0.000 | 0.000 | 0.000 | 0.000 | 0.035 | 0.199 | 0.000 | 0.000 | 0.278 | 0.001 | 0.000 | 0.189 |
| BIO02 | Mean Diurnal Range | 0.670 |  | 0.000 | 0.481 | 0.000 | 0.014 | 0.000 | 0.000 | 0.001 | 0.001 | 0.002 | 0.335 | 0.754 | 0.148 | 0.027 | 0.924 | 0.160 | 0.113 | 0.235 |
| BIO03 | Isothermality | 0.706 | 0.979 |  | 0.979 | 0.000 | 0.006 | 0.000 | 0.000 | 0.000 | 0.000 | 0.001 | 0.221 | 0.621 | 0.058 | 0.007 | 0.786 | 0.064 | 0.043 | 0.246 |
| BIO04 | Temperature Seasonality | -0.226 | 0.167 | 0.006 |  | 0.556 | 0.153 | 0.062 | 0.526 | 0.431 | 0.420 | 0.220 | 0.046 | 0.093 | 0.009 | 0.068 | 0.072 | 0.010 | 0.013 | 0.537 |
| BIO05 | Max Temp. of Warmest Month | 0.991 | 0.755 | 0.777 | -0.140 |  | 0.000 | 0.002 | 0.000 | 0.000 | 0.000 | 0.000 | 0.052 | 0.265 | 0.001 | 0.000 | 0.367 | 0.003 | 0.001 | 0.200 |
| BIO06 | Min Temp. of Coldest Month | 0.985 | 0.542 | 0.596 | -0.332 | 0.956 |  | 0.082 | 0.000 | 0.000 | 0.000 | 0.000 | 0.025 | 0.155 | 0.000 | 0.000 | 0.212 | 0.000 | 0.000 | 0.244 |
| BIO07 | Temperature Annual Range | 0.548 | 0.959 | 0.888 | 0.425 | 0.649 | 0.398 |  | 0.002 | 0.009 | 0.009 | 0.023 | 0.744 | 0.875 | 0.578 | 0.185 | 0.705 | 0.601 | 0.486 | 0.335 |
| BIO08 | Mean Temp. of Wettest Quarter | 0.912 | 0.739 | 0.754 | -0.151 | 0.915 | 0.855 | 0.648 |  | 0.000 | 0.000 | 0.000 | 0.050 | 0.168 | 0.019 | 0.017 | 0.250 | 0.025 | 0.013 | 0.037 |
| BIO09 | Mean Temp. of Driest Quarter | 0.999 | 0.684 | 0.713 | -0.186 | 0.994 | 0.979 | 0.570 | 0.912 |  | 0.000 | 0.000 | 0.043 | 0.224 | 0.001 | 0.000 | 0.312 | 0.002 | 0.001 | 0.198 |
| BIO10 | Mean Temp. of Warmest Quarter | 0.999 | 0.680 | 0.710 | -0.191 | 0.994 | 0.981 | 0.566 | 0.910 | 1.000 |  | 0.000 | 0.040 | 0.216 | 0.001 | 0.000 | 0.301 | 0.002 | 0.001 | 0.197 |
| BIO11 | Mean Temp. of Coldest Quarter | 0.998 | 0.644 | 0.690 | -0.287 | 0.983 | 0.991 | 0.506 | 0.902 | 0.994 | 0.995 |  | 0.025 | 0.164 | 0.000 | 0.000 | 0.230 | 0.001 | 0.000 | 0.184 |
| BIO12 | Annual Precipitation | -0.473 | -0.227 | -0.286 | 0.451 | -0.440 | -0.501 | -0.078 | -0.444 | -0.457 | -0.463 | -0.499 |  | 0.000 | 0.000 | 0.046 | 0.000 | 0.000 | 0.000 | 0.000 |
| BIO13 | Precipitation of Wettest Month | -0.300 | -0.075 | -0.118 | 0.386 | -0.262 | -0.330 | 0.038 | -0.321 | -0.285 | -0.289 | -0.324 | 0.958 |  | 0.003 | 0.429 | 0.000 | 0.001 | 0.001 | 0.000 |
| BIO14 | Precipitation of Driest Month | -0.709 | -0.336 | -0.431 | 0.571 | -0.674 | -0.762 | -0.132 | -0.519 | -0.693 | -0.699 | -0.740 | 0.807 | 0.625 |  | 0.000 | 0.003 | 0.000 | 0.000 | 0.111 |
| BIO15 | Precipitation Seasonality | 0.738 | 0.494 | 0.587 | -0.416 | 0.731 | 0.763 | 0.309 | 0.526 | 0.730 | 0.735 | 0.759 | -0.452 | -0.188 | -0.864 |  | 0.448 | 0.000 | 0.000 | 0.908 |
| BIO16 | Precipitation of Wettest Quarter | -0.255 | -0.023 | -0.065 | 0.411 | -0.213 | -0.292 | 0.090 | -0.270 | -0.238 | -0.243 | -0.281 | 0.955 | 0.996 | 0.624 | -0.180 |  | 0.001 | 0.001 | 0.000 |
| BIO17 | Precipitation of Driest Quarter | -0.664 | -0.327 | -0.422 | 0.563 | -0.630 | -0.712 | -0.124 | -0.500 | -0.647 | -0.654 | -0.696 | 0.856 | 0.686 | 0.992 | -0.831 | 0.687 |  | 0.000 | 0.055 |
| BIO18 | Precipitation of Warmest Quarter | -0.707 | -0.365 | -0.458 | 0.546 | -0.675 | -0.750 | -0.165 | -0.546 | -0.691 | -0.698 | -0.736 | 0.848 | 0.672 | 0.994 | -0.845 | 0.669 | 0.998 |  | 0.054 |
| BIO19 | Precipitation of Coldest Quarter | -0.307 | -0.278 | -0.272 | 0.147 | -0.299 | -0.273 | -0.227 | -0.468 | -0.300 | -0.301 | -0.310 | 0.815 | 0.897 | 0.367 | 0.028 | 0.873 | 0.436 | 0.438 |  |
